# Supplementary material for: A model for organization and regulation of nuclear condensates by gene activity
Source: Nat Commun. 2023 Jul 12;14:4152. doi: 10.1038/s41467-023-39878-4 (PMC10338548; doi:10.1038/s41467-023-39878-4)
Supplement: Supplementary file 1 — Supplementary Information [file 41467_2023_39878_MOESM1_ESM.pdf]

Supplementary Information

A model for organization and regulation of nuclear condensates by gene activity

Halima H. Schede [1,2,#], Pradeep Natarajan [2,#], Arup K. Chakraborty [2,3,4,5], Krishna Shrinivas [6,*]

[1]School of Life Sciences, École Polytechnique Fédérale Lausanne, CH-1015, Lausanne, Switzerland

[2]Department of Chemical Engineering, [3]Institute for Medical Engineering and Science, [4]Department of Physics, [5]Department of Chemistry, Massachusetts Institute of Technology, Cambridge, MA, 02139, USA

[6]NSF-Simons Center for Mathematical & Statistical Analysis of Biology, Harvard University, Cambridge, MA 02138, USA

[#]co-first authors

[*]correspondence to: krishnashrinivas@g.harvard.edu

Supplementary Information

Supplementary Table 1

Simulation parameters, predictions and biological references

Parameter / phenomenon	Model value (default)	Biological value	Reference
**[Protein] / [RNA]**	[2-200]	[5-200]	https://doi.org/10.1016/j.cell.2020.11.030 ll
**M_{rna} / $M_{protein}$**	[0.02-1.0]	[0.00025-0.3]	https://doi.org/10.1101/2021.12.31.474660 $(M_p/M_r = 0.2\ \mu m^2/s/(5\times 10^{-5}\mu m^2/s))$ = 4000 https://doi.org/10.1126/science.aar3958 $(M_p = 0.1 - 0.4\mu m^2/s)$ https://www.pnas.org/doi/pdf/10.1073/pnas.0505580102 $(M_r = 0.033\ \mu m^2/s)$
**K_p / K_d**	20.0	[0.2-64]	https://doi.org/10.1016/j.cell.2014.11.015
**Damkohler number Da = k_p * r^2 / D_{rna}**	[16-3200]	[1-100]	https://doi.org/10.1016/j.cell.2020.11.030 ll
**Flow Velocities**	$0.01 D_P/R_c$ = $0.01\ \times$ $0.5\mu m^2/s/0.4\mu m$ = 0.75 $\mu m/min$	0.15 – 1.5 um/min	https://doi.org/10.1242/jcs.226563 https://doi.org/10.1083/jcb.201904046
**Elongation and shape changes**		Condensates become	https://doi.org/10.1242/jcs.226563

		rounder when transcription is inhibited	

Parameters for simulations

Bolded letters correspond to panels of cited figure.

Supplementary Table 2: Parameters for Figure 2

Free energy	$\alpha = 0.1, \beta = 0.7, \rho_P = 1.0, \chi = 1.0, c = 10.0, \rho_R = 10.0$ and $\kappa = 0.5$
Initial conditions	**(A,B,C,D)**<br>Nucleated dense phase of protein at (0,0): $r = 4.0, \phi_P = 0.63$<br>Background protein concentration: $\phi_P = 0.12$<br>**(E,F)**<br>No nucleated dense phase of protein at (0,0).<br>Background protein concentration: $\phi_P = 0.12$
Kinetic parameters	**(A,B)** $M_p = 1.0, M_r = 1.0, k_d = 0.5, \sigma = 2.0$<br>**(C,D)** $M_p = 1.0, M_r = 1.0, k_d = 0.5, k_T = 25\ or\ 50$<br>**(E)** $M_p = 1.0, M_r = 1.0, k_d = 0.5, \sigma = 7.5$<br>**(F)** $M_p = 1.0, M_r = 1.0, k_d = 0.5$
Numerical integration	$\Delta t_{min} = 1 \times 10^{-8}, \Delta t_{max} = 0.5, total\ steps = 15000$
Geometry	2D circular domain: $r_{domain} = 30$. Mesh size: $\Delta r = 0.4$. No flux boundary condition for all species.
Parameters varied	**(A,B)**<br>$k_T = [0.1\ to\ 100]$ (for the case with gene compartment)<br>$k_T = [0.1\ to\ 1413]$ (for the uniform case)<br>**(C,D)** $\sigma = [1\ to\ 8]$<br>**(E)** $k_T = [1\ to\ 200]$<br>**(F)** $k_T = [1\ to\ 200], \sigma = [1\ to\ 8]$

Supplementary Table 3: Parameters for Figure 3

Free energy	$\alpha = 0.1, \beta = 0.7, \rho_P = 1.0, \chi = 1.0, c = 10.0, \rho_R = 10.0$ and $\kappa = 0.5$
Initial conditions	Nucleated dense phase of protein at (0,0): $r = 4.0, \phi_P = 0.63$<br>Background protein concentration: $\phi_P = 0.13$
Kinetic parameters	**(A,B)** $M_p = 1.0, M_r = 1.0, k_d = 0.5, \sigma = 3$<br>**(C)** $M_p = 1.0, M_r = 1.0, k_d = 0.5, \sigma = 4, k_T = 50$
Numerical integration	$\Delta t_{min} = 1 \times 10^{-8}, \Delta t_{max} = 0.5, total\ steps = 15000$
Geometry	2D circular domain: $r_{domain} = 30$. Mesh size: $\Delta r = 0.2$. No flux boundary condition for all species.
Parameters varied	**(A)**<br>$k_T = 1.0$ (top panel)<br>$k_T = 20.0$ (bottom panel)<br>**(B)**<br>$k_T = [1\ to\ 200]$, $\sigma = 4$ (Left panel)<br>$\sigma = [2\ to\ 8], k_T = 90.0$ (Right panel)<br>**(C)**<br>For an initial protein concentration of ϕ_{P0} at each position in space, a Gaussian noise with variance $0.1\phi_{P0}$ and zero mean was added, which resulted in the vacuoles exhibiting shape instabilities and aspherical morphologies with different centroids.

Supplementary Table 4: Parameters for Figure 4

Free energy	$\alpha = 0.1, \beta = 0.7, \rho_P = 1.0, \chi = 1.0, c = 10.0, \rho_R = 10.0$ and $\kappa = 0.5$
Initial conditions	**(A,B)** Nucleated dense phase of protein at (0,0): $r = 4.0, \phi_P = 0.63$<br>Background protein concentration: $\phi_P = 0.12$<br>**(C,D,E)** The center of the gene activity is located at distance $r = [15]$ from the nucleated dense phase of protein.
Kinetic parameters	**(A)** $M_p = 1.0, M_r = 1.0, k_d = 0.5, k_T = 10, \sigma = 5$<br>**(B,C)** $M_p = 1.0, M_r = 1.0, k_d = 0.5, k_T = 10, \sigma = 4$<br>**(D,E)** $M_p = 1.0, k_d = 0.5, k_T = 2.5, \sigma = 4$
Numerical integration	$\Delta t_{min} = 1 \times 10^{-8}, \Delta t_{max} = 0.5, total\ steps = 15000$
Geometry	**(A,D)** 2D circular domain: $r_{domain} = 30$. Mesh size: $\Delta r = 0.2$. No flux boundary condition for all species.<br>**(B)** The center of the gene activity is located at distance $r = 10$ from the nucleated dense phase of protein at (0,0)<br>**(D,E)** The center of the gene activity is located at distances $r = 15$ from the nucleated dense phase of protein.
Parameters varied	**(A)** The center of the gene activity is located at distance $r = 10$ from the nucleated dense phase of protein.<br>**(C)** The center of the gene activity is located at distances $r = [2\ to\ 25]$ from the nucleated dense phase of protein.<br>**(D)** RNA mobility M_r is varied over a range of [0.1-100]<br>**(E)** RNA degradation rate k_d is varied over a range of [0.1-2.0]

Supplementary Table 5: Parameters for Figure 5

Free energy	$\alpha = 0.1, \beta = 0.7, \rho_P = 1.0, \chi = 1.0, c = 10.0, \rho_R = 10.0$ and $\kappa = 0.5$
Initial conditions	Nucleated dense phase of protein at (0,0): $r = 4.0, \phi_P = 0.63$<br>Background protein concentration: $\phi_P = 0.13$
Kinetic parameters	$M_p = 1.0, M_r = 1.0, k_d = 0.5, \sigma = 4$
Numerical integration	$\Delta t_{min} = 1 \times 10^{-8}, \Delta t_{max} = 0.5, total\ steps = 15000$
Geometry	2D circular domain: $r_{domain} = 30$. Mesh size: $\Delta r = 0.2$. No flux boundary condition for all species.
Parameters varied	$k_T = [1\ to\ 200]$<br>The center of the gene activity is located at distances $r = [0\ to\ 25]$ from the nucleated dense phase of protein.

Supplementary Table 6: Parameters for Figure 6

Free energy	$\alpha = 0.1, \beta = 0.7, \rho_P = 1.0, \chi = 1.0, c = 10.0, \rho_R = 10.0$ and $\kappa = 0.5$
Initial conditions	Nucleated dense phase of protein at (0,0): $r = 4.0, \phi_P = 0.63$<br>Background protein concentration: $\phi_P = 0.13$<br>The distance between the sites of gene A and gene B is $r = 10$. The nucleated condensate is located at the mid point of the line joining the sites of activity.
Kinetic parameters	**(A)** $M_p = 1.0, M_r = 1.0, k_d = 0.5, \sigma_A = \sigma_B = 4$<br>**(B)** $M_p = 1.0, M_r = 1.0, k_d = 0.5, k_{TA} = k_{TB} = 10$<br>**(C)** $M_p = 1.0, M_r = 1.0, k_d = 0.5, k_{TA} = k_{TB} = 10, \sigma_A = \sigma_B = 4$
Numerical integration	$\Delta t_{min} = 1 \times 10^{-8}, \Delta t_{max} = 0.5, total\ steps = 15000$

Geometry	2D circular domain: $r_{domain} = 30$. Mesh size: $\Delta r = 0.4$. No flux boundary condition for all species.
Parameters varied	**(A)** $k_{TB} = 1, k_{TA} = [10^{-4}\ to\ 100]$<br>**(B)** $\sigma_A = 4, \sigma_B = [1\ to\ 10]$<br>**(C)** The distance between the sites of gene A and gene B is $r = [10\ to\ 24]$. The nucleated condensate is located at the mid point of the line joining the sites of activity.

Supplementary Table 7: Parameters for Figure S2

Free energy	**(A,B)** $\alpha = 0.1, \beta = 0.7, \rho_P = 1.0, \chi = 1.0, \rho_R = 10.0$ and $\kappa = 0.5$<br>**(C,D)** $\alpha = 0.1, \beta = 0.7, \rho_P = 1.0, c = 10.0, \rho_R = 10.0$ and $\kappa = 0.5$
Parameters varied	**(A,B)** $c = [0.0, 5.0, 10.0]$<br>**(C,D)** $\chi = [0.0, 1.0, 2.0]$

Supplementary Table 8: Parameters for Figure S3

Free energy	$\alpha = 0.1, \beta = 0.7, \rho_P = 1.0, \chi = 1.0, c = 10.0, \rho_R = 10.0$ and $\kappa = 0.5$
Initial conditions	Nucleated dense phase of protein at (0,0): $r = 4.0, \phi_P = 0.63$<br>Background protein concentration: $\phi_P = 0.12$
Kinetic parameters	**(A)** $M_p = 1.0, M_r = 1.0, k_d = 0.5, \sigma = 2$<br>**(B,C)** $M_p = 1.0, M_r = 1.0, k_d = 0.5$<br>**(D)** $M_p = 1.0, M_r = 1.0, k_d = 0.5, \sigma = 7.5$<br>**(E)** $M_p = 1.0, M_r = 1.0, \sigma = 4$

	**(F,H)** $M_r = 1.0, k_d = 0.5, \sigma = 4$<br>**(G)** $M_p = 1.0, k_d = 0.5, \sigma = 4$
Numerical integration	$\Delta t_{min} = 1 \times 10^{-8}$, $\Delta t_{max} = 0.5$, total steps $= 15000$
Geometry	2D circular domain: $r_{domain} = 30$. Mesh size: $\Delta r = 0.2$. No flux boundary condition for all species.
Parameters varied	**(A)** $k_T = [0.5, 0.8, 1.0, 10.0, 25.0]$<br>**(B)**<br>For exponential distribution, $k_p(x) = ce^{-\gamma x}$ with $\gamma = 1$ and 4<br>For Gaussian distribution, $k_p(x) = ce^{-\|\|x\|\|^2/2\sigma^2}$ with σ=2.0<br>**(C)** $k_T = [1\ to\ 200], \sigma = [1 \text{ to } 8]$<br>**(D)** $k_T = [1\ to\ 200]$<br>**(E)** $k_T = [0.1\ to\ 100]$ and $k_d = [0.1, 0.5, 1.0]$<br>**(F)** $k_T = [0.1\ to\ 100]$ and $M_p = [0.02, 0.1, 10.0]$<br>**(G)** $k_T = [0.1\ to\ 100]$ and $M_r = [0.02, 1.0, 50.0]$<br>**(H)** $\chi = 0$ or $c = 0$

Supplementary Table 9: Parameters for Figure S4

Free energy	$\alpha = 0.1, \beta = 0.7, \rho_P = 1.0, \chi = 1.0, c = 10.0, \rho_R = 10.0$ and $\kappa = 0.5$
Initial conditions	Nucleated dense phase of protein at (0,0): $r = 4.0, \phi_P = 0.63$<br>Background protein concentration: $\phi_P = 0.13$
Kinetic parameters	**(A)** $M_P = 1.0, M_R = 1.0, k_d = 0.5$<br>**(B,C)** $M_P = 1.0, M_R = 1.0, k_d = 0.5, \sigma = 4, k_T = 50$<br>**(D)** $M_P = 1.0, M_R = 1.0, k_d = 0.5, \sigma = 3, k_T = 50$<br>**(E)** $M_P = 1.0, M_R = 1.0, k_d = 0.5, \sigma = 2, k_T = 50$<br>**(F)** $M_p = 1.0, M_r = 1.0, \sigma = 4$<br>**(G)** $M_p = 1.0, k_d = 0.5, \sigma = 4$<br>**(H)** $M_r = 1.0, k_d = 0.5, \sigma = 4$<br>**(I)** $M_r = 1.0, M_r = 1.0, k_d = 0.5, \sigma = 4$
Numerical integration	$\Delta t_{min} = 1 \times 10^{-8}, \Delta t_{max} = 0.5, total\ steps = 15000$
Geometry	2D circular domain: $r_{domain} = 30$. Mesh size: $\Delta r = 0.2$. No flux boundary condition for all species.
Parameters varied	**(A)** $k_T = [1\ to\ 200]$, $\sigma = 4$ (Left panel)<br>$\sigma = [2\ to\ 8], k_T = 90.0$ (Right panel)<br>**(B,C)** For an initial protein concentration of ϕ_{P0} at each position in space, a Gaussian noise with variance 0.1ϕ_{P0} and zero mean was added, which resulted in the vacuoles exhibiting shape instabilities and aspherical morphologies with different centroids.<br>**(D)** $\kappa = [0.5\ to\ 2.0]$ (for $k_T = 30$)<br>$\kappa = [0.01\ to\ 0.4]$ (for $k_T = 50$)<br>**(E)** $M_R = [0.1\ to\ 10]$

	**(F)** $k_T = [1\ to\ 500]$ and $k_d = [0.1, 0.5, 1.0]$<br>**(G)** $k_T = [1\ to\ 500]$ and $M_r = [0.1, 1, 10]$<br>**(H)** $k_T = [1\ to\ 500]$ and $M_p = [0.02, 1, 10]$<br>**(I)** $k_T = [1\ to\ 500]$ and $c = 0$ or $\chi = 0$

Supplementary Table 10: Parameters for Figure S5

Free energy	$\alpha = 0.1, \beta = 0.7, \rho_P = 1.0, \chi = 1.0, c = 10.0, \rho_R = 10.0$ and $\kappa = 0.5$
Initial conditions	Nucleated dense phase of protein at (0,0): $r = 4.0, \phi_P = 0.63$<br>Background protein concentration: $\phi_P = 0.13$<br>**(A)** The center of the gene activity is located at distance $r = [10]$ from the nucleated dense phase of protein.<br>**(B)** Nucleated dense phase of protein at (0,0)<br>**(C,D)** The center of the gene activity is located at distance $r = [15]$ from the nucleated dense phase of protein.
Kinetic parameters	**(A)** $M_P = 1.0, k_d = 0.5, k_T = 2.5, \sigma = 4$<br>**(B)** $M_P = 1.0, M_r = 1.0, k_d = 0.5, k_T = 2.5, \sigma = 4$<br>**(C,D)** $M_P = 1.0, M_R = 1.0,\ k_d = 0.5, k_T = 10, \sigma = 4$
Numerical integration	$\Delta t_{min} = 1 \times 10^{-8}, \Delta t_{max} = 0.5, total\ steps = 15000$
Geometry	2D circular domain: $r_{domain} = 30$. Mesh size: $\Delta r = 0.2$. No flux boundary condition for all species.
Parameters varied	**(A)** $M_R = [0.01, 1.0, 100.0]$<br>**(B)** The center of the gene activity is located at distance $r = [2, 8, 12, 14, 20]$ from the nucleated dense phase of protein.<br>**(C,D)** $\kappa = [0.01\ to\ 0.5]$

Supplementary Table 11: Parameters for Figure S6

Free energy	**(A,B,C)** $\alpha = 0.1, \beta = 0.7, \rho_P = 1.0, \chi = 1.0, c = 10.0, \rho_R = 10.0$<br><br>**(D)** $\alpha = 0.1, \beta = 0.7, \rho_P = 1.0, \chi = 1.0, c = 10.0, \rho_R = 10.0, \kappa = 0.05$
Initial conditions	Nucleated dense phase of protein at (0,0): $r = 4.0, \phi_P = 0.63$<br><br>Background protein concentration: $\phi_P = 0.13$
Kinetic parameters	**(A,B,C,D)** $M_P = 1.0, M_R = 1.0, k_d = 0.5, k_{TA} = k_{TB} = 1, \sigma_A = \sigma_B = 4$
Numerical integration	$\Delta t_{min} = 1 \times 10^{-8}, \Delta t_{max} = 0.5, total\ steps = 15000$
Geometry	**(A,B,C)** 2D circular domain: $r_{domain} = 30$. Mesh size: $\Delta r = 0.2$. No flux boundary condition for all species.<br><br>**(D)** The distance between the centers of gene A and gene B was fixed at $r/2\sigma = 2.5$
Parameters varied	**(A)** $\kappa = [0.005\ to\ 0.5]$<br><br>The distance between the centers of gene A and gene B was varied from $r/2\sigma = [0\ to\ 2.5]$<br><br>**(B,C)** The distance between the centers of gene A and gene B was varied from $r/2\sigma = [1, 1.5, 2, 2.5]$<br><br>**(D)** $\kappa = [0.01, 0.05, 0.1, 0.5]$

a

Free energy

$f\,[\varphi_p(x,t),\ \varphi_r(x,t)]$

$= \int_V dx\ \{\ (f_{dw}(\varphi_p) + \rho_r\varphi_r^2)$ **protein-protein & RNA-RNA interactions**

$+\ \kappa/2\ (\nabla\varphi_p)^2$ **surface tension**

$+\ \chi_{eff}(\varphi_p,\ \varphi_r)\ \}$ **protein-RNA interactions**

$\chi_{eff}(\varphi_p,\ \varphi_r) = -\ \chi\varphi_p\varphi_r + c\varphi_p^2\varphi_r^2$

b

Dynamics

protein dynamics

$$\partial_t\varphi_p(x,t) = \underbrace{\nabla(M_p\nabla(df/d\varphi_p))}_{\text{diffusion}}$$

RNA dynamics

$$\partial_t\varphi_r(x,t) = \underbrace{\nabla(M_r\nabla(\varphi_r))}_{\text{diffusion}} + \underbrace{k_p(x)\ \varphi_p}_{\text{RNA synth.}} - \underbrace{k_d\varphi_r}_{\text{degradation}}$$

$$k_p(x) = k_p\ e^{-||x - x0||^2/(2\sigma^2)}$$

c

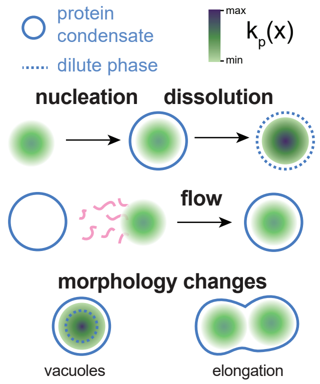

Supplementary Figure 1. Model equations of active nuclear condensates predicts protein dynamics. **(a)** Free energy equations. f: free energy functional; x: position in space; t: time; φ_p: protein concentration; φ_r: RNA con centration; f_{dw}: double-well potential capturing phase separation of protein due to protein-protein interactions; ρ_r: RNA-RNA interaction strength; κ: surface tension **(b)** Dynamic equations for concentrations of protein and RNA species. The free energy and dynamic equations are similar to Henninger et al. (2021), with the key difference being the spatially varying gene activity $k_p(x)$ **(c)** Predictions from the model in line with various biological observations including nucleation, dissolution, directed motion and morphology changes including vacuoles and elongation.

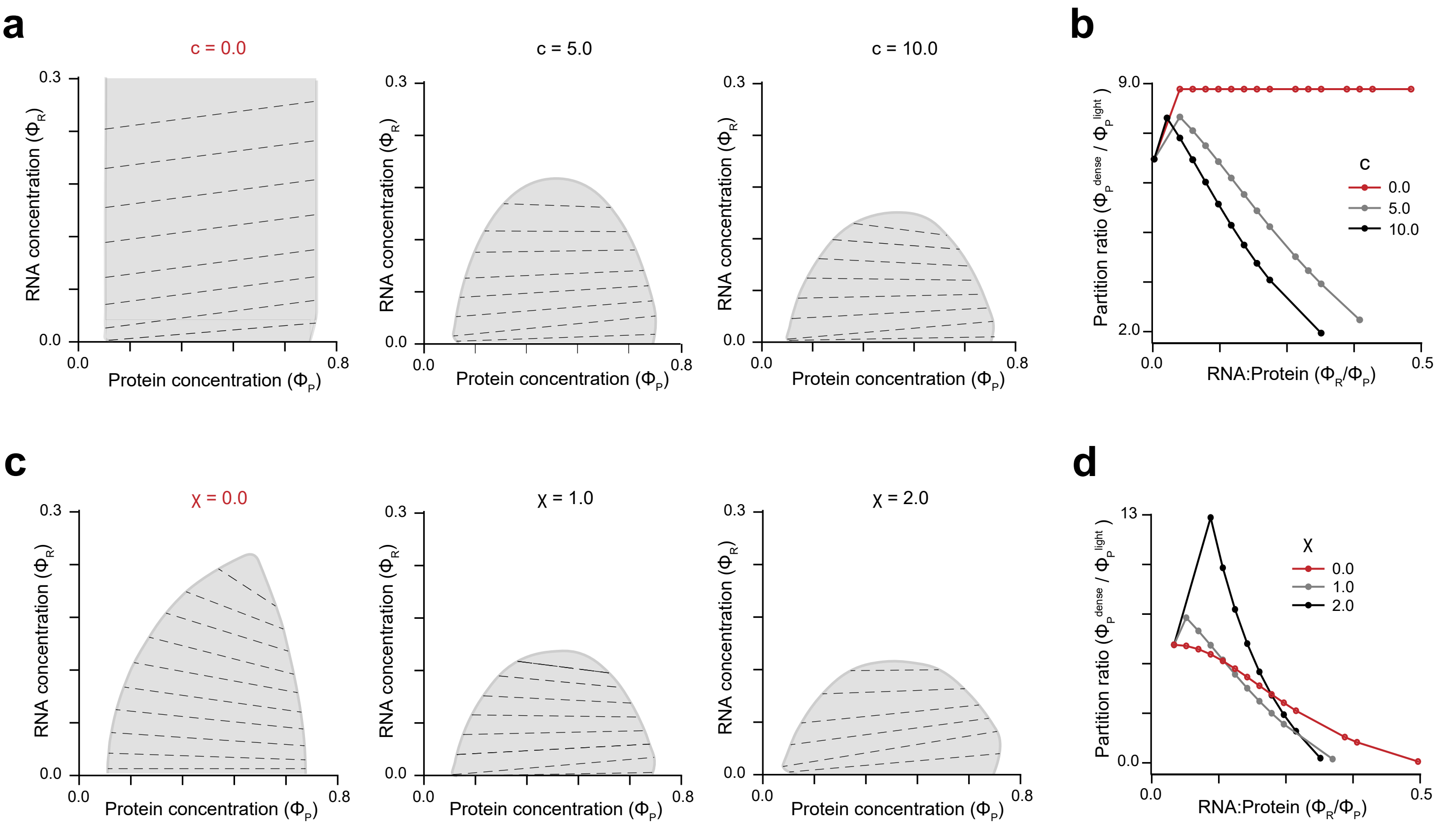

Supplementary Figure 2. The free-energy functional used to model active nuclear condensates is parameter ized to reflect biological observations for re-entrant phase behavior. (a) Phase diagrams for various values of free energy parameter **c**. The binodal curve encompassing the 2-phase region is depicted in grey. The black dotted lines are tie-lines indicating the direction of phase separation in the 2-phase region. The right boundary of the binodal curve corresponds to the dense phase of protein and the left boundary corresponds to the dilute pha se. **(b)** Re-entrant phase behavior for various values of free energy parameter **c**. The partition ratio of protein (φ_p^{dense} / φ_p^{dilute}) is plotted as a function of the RNA:Protein ratio (φ_r/φ_p). For this plot, φ_r is increased keeping φ_p at a constant value of 0.4. **(c)** Phase diagrams for various values of free energy parameter **χ**. **(d)** Re-entrant phase behavior for various values of free energy parameter **χ**. The partition ratio (φ_p^{dense} / φ_p^{dilute}) is plotted as a function of the RNA:Protein ratio (φ_r/φ_p). φ_r is increased keeping φ_p at a constant value of 0.4.
Please refer to Supplementary Table 7 for details of simulation parameters.

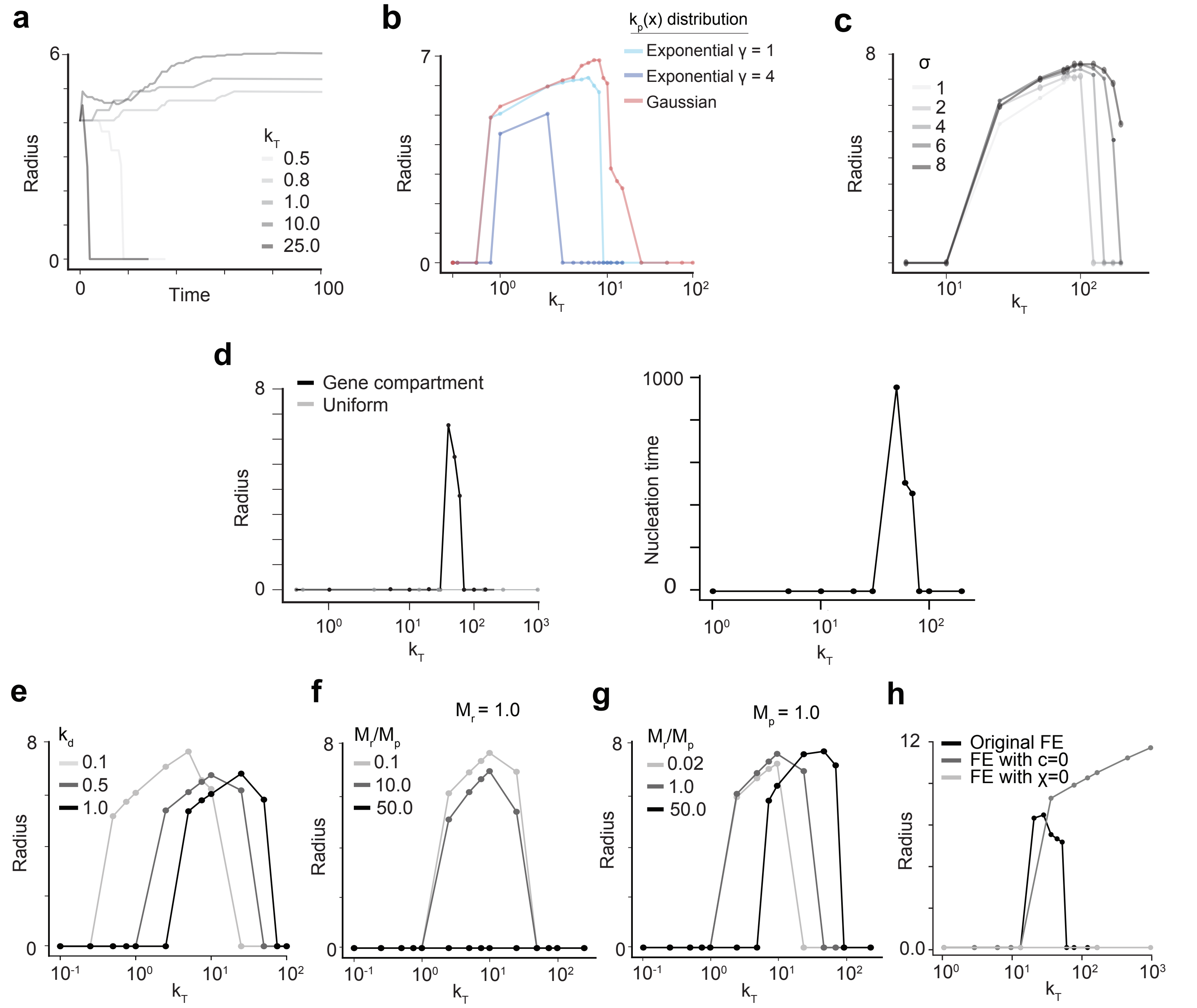

Supplementary Figure 3. Effect of different parameters on condensate size and nucleation.

(a) Dynamics condensate radius for different values of k_T values over time. **(b)** Effect of different functional forms of spatially varying gene activity $k_p(x)$. Plotted are steady-state condensate radii as a function of the total RNA activity (k_T) where $k_p(x)$ is either an exponential distribution with $\gamma = 1$ or $\gamma = 4$, or a Gaussian distribution which is used for the bulk of this study. **(c)** Steady-state condensate radii vs k_T for different values of compartmentalization (σ). **(d)** The left panel describes the impact of k_T on condensate radius when there is no initially nucleated condensate, for spatially clustered and uniform RNA production. The right panel show the time required to nucleate a dense phase of protein for different values of k_T **(e)** Steady-state condensate radii vs k_T for various RNA degradation rates (k_d) **(f)** Steady-state condensate radii vs k_T for various ratios of RNA/Protein diffusivity (M_r/M_p). These plots are generated by varying M_p while fixing $M_r = 1.0$. **(g)** Steady-state condensate radii vs k_T for various ratios of RNA/Protein diffusivi ty (M_r/M_p). These plots are generated by varying M_r while fixing $M_p = 1.0$. **(h)** Steady-state condensate radii vs k_T using the original free energy expression and comparison with parameter values for free energy, $c = 0$ and $\chi = 0$, that are inconsistent with a re-entrant phase transition. With $c = 0$, we do not get condensate dissolution upon increasing k_T. With $\chi = 0$, we do not have condensate nucleation as well. Please refer to Supplementary Table 8 for details of sim ulation parameters.

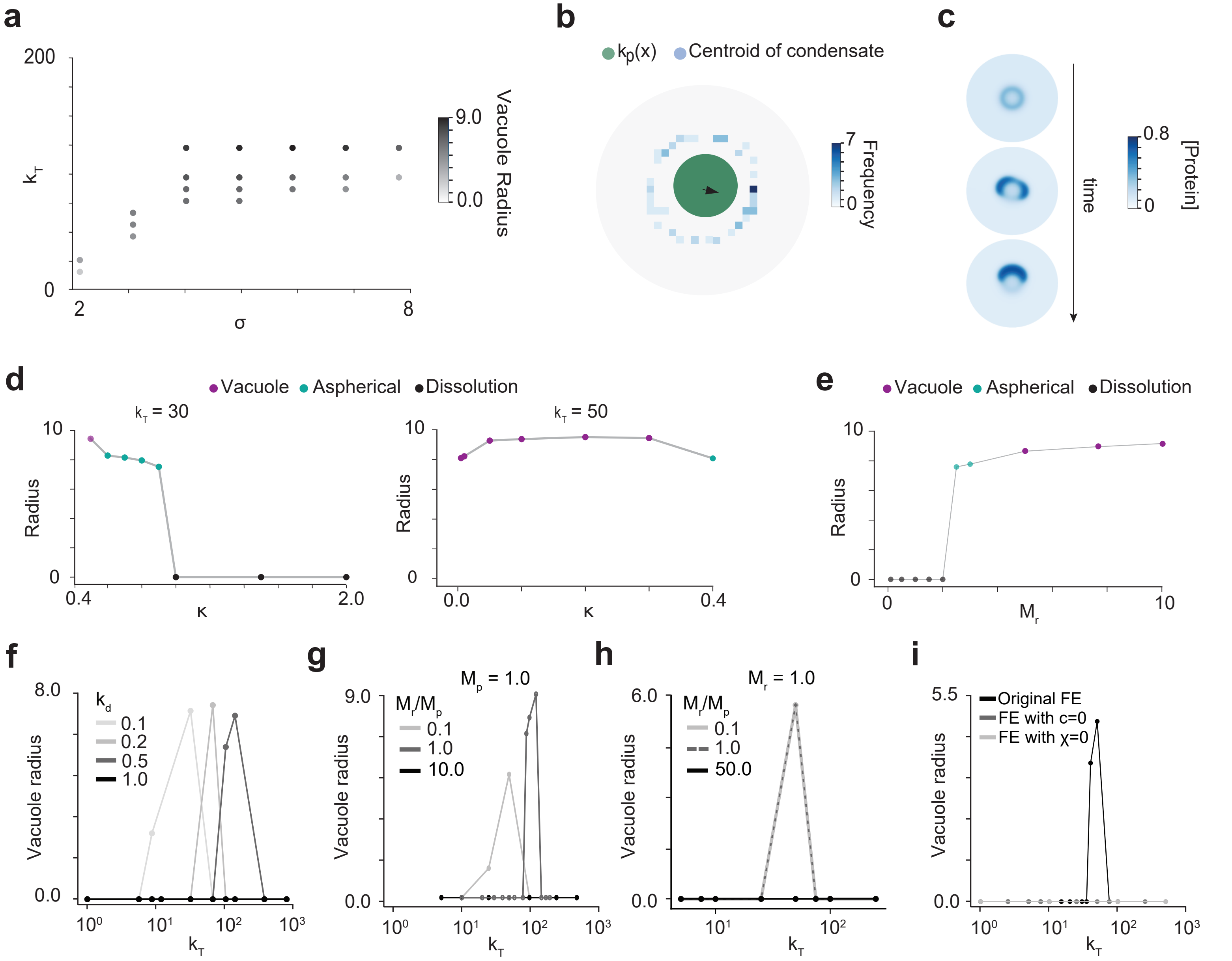

Supplementary Figure 4. Non-equilibrium steady-state morphologies are modulated by surface tension and RNA mobility (a) Vacuole radius (color of dot) for different values of gene activity (k_T) and extent of compartmentalization. **(b)** Histogram of centroids of aspherical droplets formed after symmetry-breaking for different initial conditions. There is no preferred direction for the centroid of the aspherical droplet. **(c)** In few simulations, symmetry-breaking initially leads to two aspherical condensates that eventually fuse into one at steady-state. **(d)** Role of surface tension in symmetry breakage and vacuole formation. Left: at lower values of k_T, increasing surface tension (κ) causes a vacuole to break symmetry and eventually dissolve. Right: For larger k_T decreasing surface tension (κ) causes a condensate which initially broke symmetry to stabilize and form a vacuole. **(e)** The effect of RNA mobility on condensate morphologies at steady state. Left: condensate radii at steady state as a function of RNA mobility coefficient. Increasing mobility decrease s local RNA concentration, which leads to condensate stabilization, first in an aspherical droplet, then to a symmetric vacuole. **(f)** Vacuole radius as a function of k_T for various rates of RNA degradation (k_d). Increasing the RNA stability by decreasing k_d results in vacuoles formation at lower k_T **(g) & (h)** Vacuole radius as a function of k_T for various ratios of RNA to protein mobility (M_r/M_p) while keeping M_p = 1.0 in (g) and M_r = 1.0 in (h). In both cases, there is no vacuole formation when M_r/M_p>> 1 **(i)** Vacuole radii using the original free energy expression and comparison with parameter values for free energy, c= 0 and χ = 0, that are inconsistent with a re-entrant phase transition. In both these cases, there is no vacuole formation. Please refer to Supplementary Table 9 for details of simulation parameters.

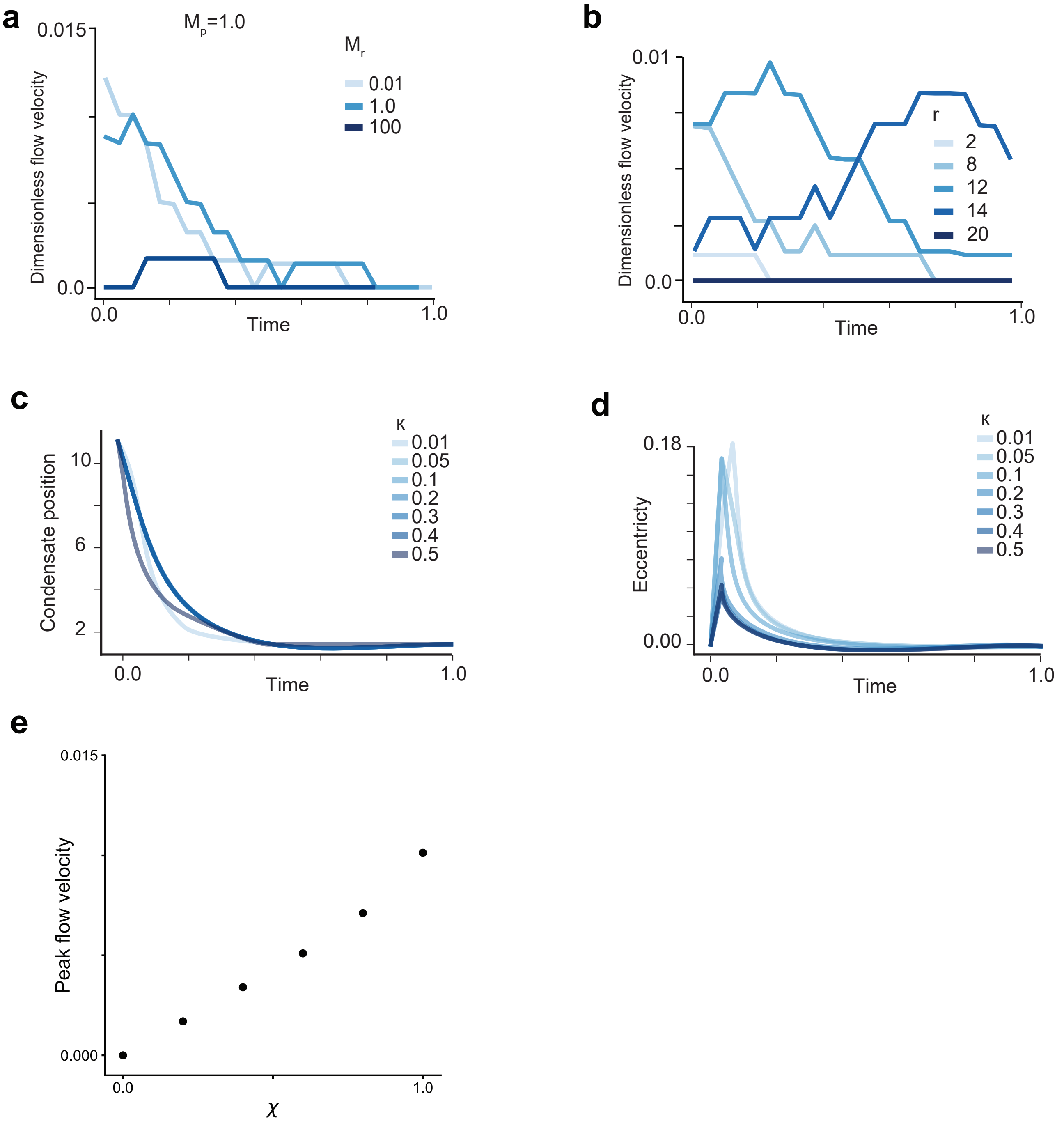

Supplementary Figure 5. Flow of nuclear condensates is dependent on RNA mobility, initial distanc e between the condensate and gene locus and surface tension.

(a) Flow velocity of the condensate over time with various values of RNA mobility (M_r). **(b)** Flow velocity of the condensate over time from various starting distances between condensate and gene locus (r). **(c)** Change in condensate position over time under different sets of surface tension (κ). **(d)** Eccentricity (which measures condensate deformation) over time under different sets of surface tension (κ). An eccentricity of 0 corresponds to a perfectly round condensate. **(e)** The peak flow velocity of condensates scales linearly with the RNA-protein attraction strength (χ) and becomes 0 when χ=0, indicating that this is the interaction that drives flow. Please refer to Supplementary Table 10 for details of simulation parameters.

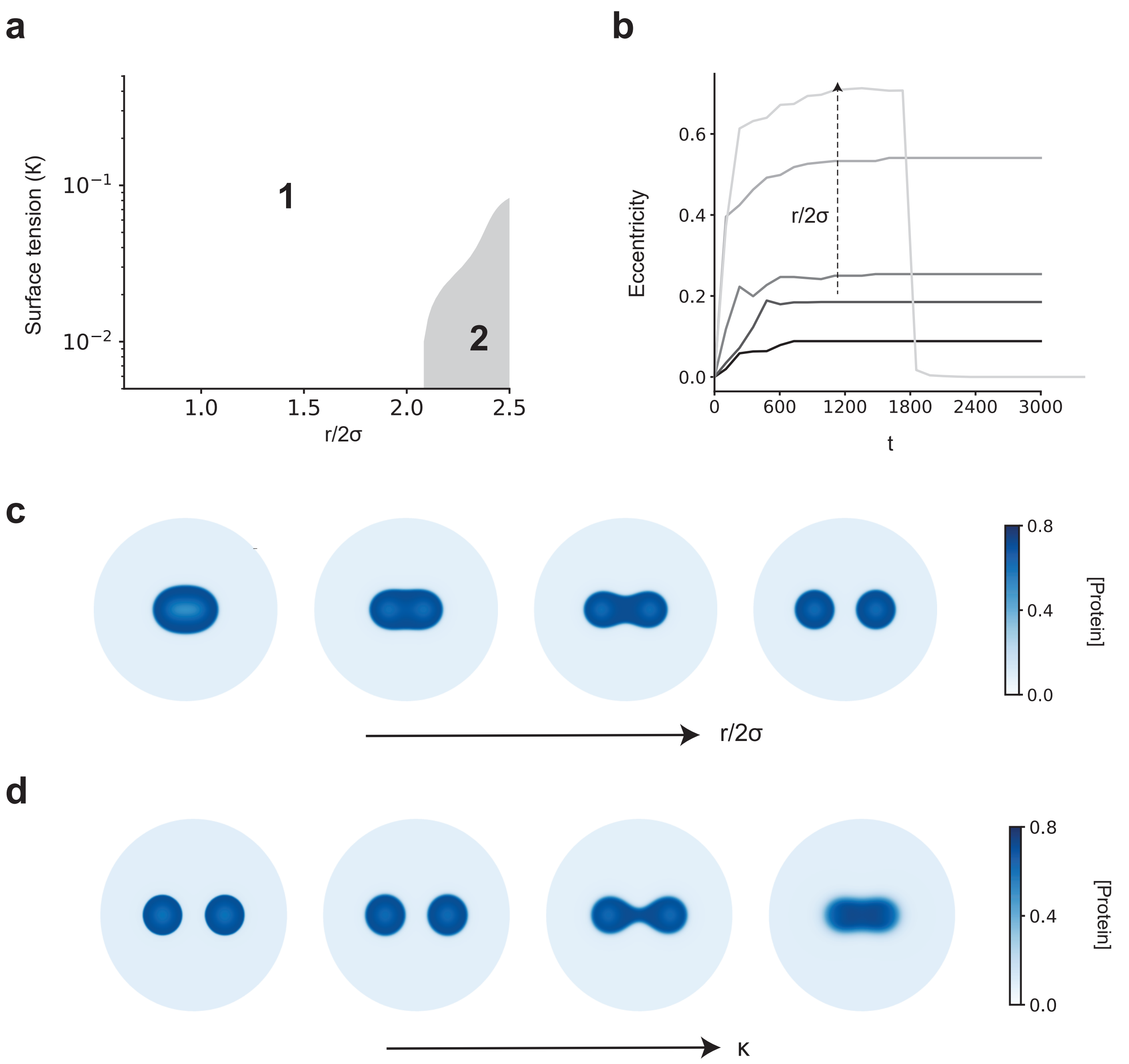

Supplementary Figure 6. Two sites of activity can lead to droplet division.

(a) Phase diagram for condensate morphology upon varying the surface tension of the protein (κ) and the distance between two active genomic loci, r. The distance between the loci is reported as a dimensionless ratio with the width of the genomic locus (r/2σ). In the parameter regime 1 - we have a single droplet at steady state. In parameter regime 2 where the surface tension is low and the loci are far apart, the system at steady state has two droplets each centered around one of the loci. **(b)** Dynamics of the eccentricity of the droplets for different distances between the loci (r/2σ). As the distance between the loci increases, the eccentricity of the droplet increases more rapidly as the droplet reaches a deformed steady state shape. Beyond a threshold distance (light grey), the single droplet deforms so much after a certain time that it quickly splits into two spherical droplets which is a more energetically favorable configuration. **(c)** Steady state droplet morphology upon varying distance between the loci. Increasing the distance between the loci leads to a gradual deformation of the droplet. Beyond a threshold distance the droplet splits into two, each one centered around one of the loci. **(d)** Steady state droplet morphology upon varying the surface tension (κ).

Increasing the surface tension penalizes interface formation and progressively favors a single droplet configuration. Please refer to Supplementary Table 11 for details of simulation parameters.
